# Development of an mRNA vaccine against the OprF of *Pseudomonas aeruginosa* and evaluation of antibody response in mouse

**DOI:** 10.1128/spectrum.00473-25

**Published:** 2026-08-12

**Authors:** Masoumeh Fathi, Lida Lotfollahi, Fatemeh Mehravar, Bizhan Nomanpour

**Affiliations:** 1 Department of Microbiology, School of Medicine, Kermanshah University of Medical Sciences 48464, Kermanshah, Iran; 2 Department of Microbiology and Virology, School of Medicine, Urmia University of Medical Sciences, Urmia, Iran; 3 Department of Public Health, Golestan University of medical Science, Gorgan, Iran; Tainan Hospital Ministry of Health and Welfare, Tainan, Taiwan

**Keywords:** *Pseudomonas aeruginosa*, antibiotic resistance, lipid nanoparticles, mRNA vaccine

## Abstract

Recently, antibiotic resistance has been recognized as a major threat to global public health. *Pseudomonas aeruginosa* (PA) is one of the most important opportunistic pathogens that mainly lead to dangerous nosocomial infections occurring in immunocompromised individuals. In recent years, with the increasing rate of multidrug resistance (MDR) of PA, management of infection with this pathogen has become a global challenge. Messenger RNA (mRNA) vaccines seem to be a good alternative to conventional vaccines. In the present study, an mRNA vaccine encoding OprF—outer membrane protein F—in PA was produced and synthesized using the *in vitro* transcription (IVT) method. Additionally, lipid nanoparticles (LNPs) were prepared by a thin-layer hydration method, and the prepared mRNA-LNP vaccine was evaluated in terms of nanoparticle properties. Finally, the efficacy of the vaccine in inducing a humoral immune response against PA was studied in BALB/c mice through injecting 5 or 25 μg of mRNA to each group. ELISA results showed an increase in the level of neutralizing antibodies against OprF protein in PA. Immunization with mRNA-OprF induced an appropriate humoral immune response and reduced bacterial burden in systemic infection models. It seems that the mRNA-OprF vaccine made in the present study can be a promising vaccine to prevent PA infection.

**IMPORTANCE:**

This work is one of the suitable mRNA-LNP vaccine designs because it targets an important immunogenic antigen that can induce both humoral and adaptive immunity against bacteria. As the first project of mRNA vaccine, especially for a non-viral microorganism in Iran, our results demonstrate good humoral immunity Induced by the vaccine developed against *P. aeruginosa*. This work is of great importance and has significant potential to provide and develop an efficient vaccine against *P. aeruginosa* infections.

## INTRODUCTION

Antimicrobial resistance (AMR) is recognized as an important global concern and continues to increase (1). The importance of this matter is so great that AMR was introduced by the World Health Organization (WHO) as one of the 10 major threats to global health in 2019 (2). In the United States, at least 2.8 million new cases of antibiotic-resistant infections occur each year, with at least 35,000 deaths from the phenomenon, according to the Centers for Disease Control and Prevention (CDC) (3). The incidence of AMR infections seems to be increasing since 2007 (4). According to the reported statistics, at least 700,000 people die from AMR every year, and if this trend continues, the number of deaths caused by AMR is expected to increase to 10 million people per year by 2050 (5). In recent years, with the irrational and excessive use of broad-spectrum antibiotics, drug resistance has increased among bacteria (6). On February 27, 2017, in order to facilitate the focus and guide the development of new and effective antibiotics, the WHO published its first list of antibiotic-resistant pathogens, called ESKAPE, which appear to be a major threat to health. This list is determined based on “priority status,” and the development of new antibiotics against these pathogens is necessary (7, 8). *P. aeruginosa* is a gram-negative opportunistic pathogen and an important nosocomial pathogen, especially in patients admitted to burn care and intensive care units (ICU) (9, 10). Because the antibiotic resistance of this bacterium has increased significantly in recent years, the treatment and management of related infections has become one of the most challenging therapeutic phenomena (11). Therefore, it seems that to deal with the growing problem of antibiotic resistance in resistant and life-threatening pathogens, it is necessary to design a series of strategies at the global level. Recently, vaccines have been introduced as an appropriate approach to deal with antibiotic-resistant pathogen infections, including *P. aeruginosa* (12, 13). Despite more than 50 years of research and efforts to develop effective vaccines to combat *Pseudomonas* infections, there is still no licensed vaccine on the market for this pathogen (14). Outer membrane protein F (OprF) has been widely investigated as a target antigen in *P. aeruginosa*, and it seems that this antigen is highly conserved and immunogenic in all *P. aeruginosa* strains (15–19). Compared to conventional vaccines, mRNA vaccines are a promising alternative due to their low cost, rapid development, safety, efficacy, capacity to induce better immune response, and lack of genomic integrity risk (14, 20, 21). The success of these vaccines in combating viral infections has led researchers to consider designing these vaccine platforms against bacterial infections. The number of studies on the use of these vaccines against bacterial pathogens is limited. However, studies on bacteria such as *Mycobacterium tuberculosis*, *Bordetella pertussis*, and *P. aeruginosa* are being developed to investigate the effectiveness of RNA vaccines against these infections. Some of these studies have shown promising results in the development of mRNA vaccines against bacterial pathogens. However, it should be noted that the development of mRNA vaccines against bacterial pathogens is in its infancy (14, 22). Consequently, in this study, we designed an mRNA vaccine construct against OprF in *P. aeruginosa*. After *in vitro* transcription, lipid nanoparticles (LNPs) were prepared through a common liposome preparation method called thin-film hydration method. Then, the immunogenicity and protective effect of the mRNA-LNP vaccine were evaluated in BALB/c mice.

## MATERIALS AND METHODS

### Bacterial strains and mice

*P. aeruginosa* standard strain PAO1 (from the microbial collection of the Department of Medical Microbiology, Urmia University of Medical Sciences), *Escherichia coli* Top10 F′, and *E. coli* BL21 (DE3) were used in this study. Four- to 6-week-old female BALB/c mice (20–30 g) were purchased from the Laboratory Animal Production Department at Urmia University of Medical Sciences (Urmia, Iran). All the mice were kept under pathogen-free conditions and under a 12-hour light/dark cycle and were fed a normal diet with adequate water. All mice were monitored daily during the experiments. In this study, all animal experiments were performed in accordance with the guidelines of the Declaration of Helsinki and were approved by the Research Ethics Committee of the Ministry of Health, Medicine and Medical Education of Iran (IR.KUMS.AEC.1401.020).

### PCR amplification and cloning of the *oprF* gene

The sequence of the *oprF* gene was extracted from the valid databases (https://ncbi.nlm.nih.gov/gene/878442). Primers for the *oprF* gene (complete sequence) were designed based on the *oprF* gene sequence of PAO1 (Table 1). In addition, restriction sites of EcoRI and HindIII enzymes were placed at the 5′ end of both forward and reverse primers, respectively. Genomic DNA extraction was performed according to the protocol of the kit (Favorgen, Taiwan). Amplification of our desired gene was performed using *Pfu* DNA polymerase (Fermentas, Lithuania). The amplification was briefly performed according to the following steps: after predenaturation at 95°C for 5 min, 30 cycles were performed at 95°C for 1 min, 54°C for 1 min, and 72°C for 1 min, followed by a final extension at 72°C for 10 min. PCR products of the *oprF* gene were purified by a purification kit (Favorgen, Taiwan) and analyzed by DNA agarose gel electrophoresis. Cloning of the *oprF* gene was carried out by ligation of the purified PCR product into the GetClone PCR Cloning Vector II according to the manufacturer’s protocols (Smobio, Taiwan). The ligation prepared was transformed into the *E. coli* Top10 F′*,* and screening was performed by colony PCR (23). Positive clones were sequenced (BioNeer, South Korea) for analysis of the sequence integrity.

**TABLE 1:** Primer sequence used in *oprF* gene amplification

Gene	Primer sequences	Restriction enzymes	Reference
*oprF*	**F:GAATTC** GTTGTGCGGCTGATTGTTGGAC	EcoRI	This study
	**R:AAGCTT** GAGGCTCAGCCGATTACTTGG	HindIII	This study

### Design and synthesis of mRNA vaccine construct

During the current experiment, 5′ untranslated region (UTR), Kozak sequence, and 3′ UTR region were designed (14). Synthesis of the sequence was performed by Gene Universal Company (Canada), and the entire sequence was cloned into the pBluescript SK (+) vector.

### Subcloning of the *oprF* gene in chemically synthesized construct for mRNA vaccine

For synthesis of the OprF mRNA, *oprF* insert was digested and ligated into the EcoRI–HindIII sites of the pBluescript SK (+) vector. The ligation product was transformed into the *E. coli* Top10 F′ via the heat-shock method. The construct was subsequently verified by colony PCR and DNA sequencing (23).

### Preparation of linear plasmid

A single colony containing the pBluescript SK (+)-*oprF* plasmid was added to LB broth containing ampicillin (100 µg/mL) and incubated at 37°C for 12–15 h with shaking at 180 rpm. After collecting the bacterial cells by centrifugation, plasmid extraction was performed according to the protocol of the plasmid DNA extraction kit (Favorgen, Taiwan). Then, the XbaI enzyme cleavage site of the amplified plasmid was selected for plasmid linearization. Through agarose DNA gel electrophoresis, the degree of linearization of the desired plasmid was determined. After purification of the linearized plasmid by PCR product recovery kit (Favorgen, Taiwan), DNA quality and concentration were determined by NanoDrop (BioTek, USA) (14).

### *In vitro* transcription and purification of mRNA

*In vitro* mRNA synthesis was performed via T7 polymerase-mediated DNA transcription. *In vitro* mRNAs were synthesized according to the manufacturer’s protocol of mMESSAGE mMACHINE T7 Ultra Kit (Thermo Fisher Scientific, USA). After extracting the synthesized mRNAs by phenol-chloroform method, finally, the concentration and quantity of transcribed mRNAs were calculated using NanoDrop (BioTek, USA).

### mRNA-LNPs preparation

Preparation of liposomes containing mRNA was performed using a thin layer hydration method. In brief, first, soy lecithin (4 mg) was dissolved in chloroform (0.5 mL), and then, the organic solvent was evaporated using a rotary evaporator (HB 10 S099 instrument, IKA, Germany). The synthesized mRNA was dissolved in 10 mL of distilled water (35°C) and then added to the obtained lipid film and hand-shaken at room temperature. The suspension solution contained multilayered liposomes. Finally, the size of liposomes was reduced using an ultrasound probe (UW2070, BANDELIN electronic, Germany) at 85 W for five cycles of 5 min with a resting period of 5 min. The solution was placed in a bath sonicator (D-78224, Elma, Germany) for 55 min (24).

### Determination of mRNA-LNPs characterization

The particle size and polydispersity index (PDI) of LNPs obtained were measured using dynamic light scattering (DLS) (Malvern Instruments Ltd Worcestershire, UK). In addition, zeta potential was also measured with the same instrument (24).

### Determination of encapsulation efficiency and mRNA loading

The amount of unentrapped mRNA in the supernatant obtained by ultracentrifugation of the liposome at 30,000 rpm for 30 min was assayed by NanoDrop spectrophotometry at an absorbance ratio of 260/280 nm. Therefore, the amount of trapped mRNA was accounted via subtracting the unencapsulated amount from the total amount of mRNA added to the formulation. The amount of drug encapsulated in hydrated liposomes is considered as the loading capacity (24). The mRNA loading and entrapment efficiency (EE) were calculated using the following equations:


$$\begin{array}{l} \text{Encapsulation}\%=\frac{\text{mRNA free}-\text{Total mRNA}}{\text{Total mRNA}}\\ \text{Drug loading}\%=\frac{\text{mRNA free}-\text{Total mRNA}}{\text{Total mRNA}+\text{carrier weight}} \end{array}$$

### Evaluation of the stability and shelf life of mRNA-containing lipid nanoparticles (LNPs)

In order to investigate the shelf life and stability of lipid nanoparticles (LNPs) containing mRNA, three parameters, including particle size, polydispersity index (PDI), and zeta potential of LNPs, were investigated using dynamic light scattering (DLS) after storage in a refrigerator (5°C) (for 30 days) and room temperature (25°C) (0, 1, 2, 4, 24, and 48 h).

### OprF protein expression and purification

In order to achieve recombinant expression of OprF protein in *E. coli*, the *oprF* insert in the GetClone PCR Cloning Vector II was digested and ligated into the EcoRI–HindIII sites of the pET-28a (+) expression vector. Then, the pET-28a (+)-*oprF* construct was transformed into *E. coli* BL21 (DE3) strain (Novagen, Darmstadt, Germany) via the heat-shock method. This strain was cultured in LB broth containing 50 µg/mL kanamycin (Sigma-Aldrich, Germany) and incubated at 37°C. At an optical density of 0.6 (600 nm), 1 mM IPTG was added, and the culture continued for another 3 h. After collecting the cells by centrifugation at 6,000 × *g* for 3 min, protein expression was evaluated using SDS-PAGE. After ensuring protein expression, the cells were crushed using a high-pressure homogenizer, and the supernatant obtained after high-speed centrifugation was purified by Ni-NTA affinity chromatography (Qiagen, USA) (25).

### Vaccination experiments of mice

In the present study, all stages of animal experiments, including blood sampling, anesthesia, and euthanasia, were performed according to the guidelines of the Research Ethics Committee of the Ministry of Health, Medicine and Medical Education of Iran (ethics registration code: IR. KUMS.AC.1401.020). To determine the immune responses of mRNA-OprF-LNP *in vivo*, 21 BALB/c mice were randomly divided into three groups (seven mice in each group). Two groups of mice were injected intramuscularly (I.M.) with 100 μL of mRNA-LNP containing 5 or 25 μg of mRNA encoding OprF. The control group received LNP only. Subsequently, 2 weeks after the first immunization (14 days), the second booster vaccination was performed with the same dose and volume. Blood samples were collected on days 14 and 21 after the first injection of the vaccine and on day 14 after the second injection. Blood sampling was done under mixed ketamine–xylazine anesthesia (100 mg/kg ketamine and 5 mg/kg xylazine). This injection was done once intraperitoneally. The average duration of anesthesia was 30 min. Blood samples were collected from the orbital venous plexus of all rats. A person specialized in working with laboratory animals supervised all stages of anesthesia and blood sampling in the animals. Serum was separated by centrifugation at 3,000 rpm for 20 min and stored at −80°C until analysis. At the end of the study, a safe and irreversible euthanasia method was used, and a location away from other animals was chosen. Euthanasia was performed intraperitoneally by injecting sodium pentobarbital with a concentration of 60 mg/mL from a sodium thiopental solution at a dose of 20 mg per 100 g of animal body weight (14, 26, 27).

### Determination of mouse-specific antibody titers via ELISA

In this study, we measured the specific titer of anti-OprF IgG in serum using an indirect enzyme-linked immunosorbent assay (ELISA) according to the following steps. Briefly, the blank 96-well ELISA plates were coated with OprF protein expressed in *E. coli* and incubated overnight at 4°C; then the plates were washed three times with PBST (phosphate-buffered saline containing 0.05% Tween-20) and blocked with 1% BSA at 37°C for 2 h. After three washes, each well was incubated at 37°C for 1 h after adding 100 μL of diluted mouse serum. After another three washes, 100 μL of HRP-labeled rabbit anti-mouse IgG (diluted 1:1,000) was added, and the plates were incubated at 37°C for 1 h. After three washes, 100 μL of HRP chromogenic substrate TMB was added and incubated in the dark at room temperature for 15 min. Finally, to stop the reaction, 100 μL of 2% sulfuric acid was added, and the OD450 of the absorbance was measured (14, 26).

### Protective efficacy against *P. aeruginosa* challenge

For the bacterial challenge, 2 weeks after the last injection, all mice in all three groups were infected intraperitoneally with a dose of 10 × LD50 of PAO1 strain of *P. aeruginosa*. The challenged mice were examined and observed over 5 days.

For the bacterial challenge, 2 weeks after the last injection, all the mice were infected at a dose of 10 × LD_50_ of the PAO1 strain of *P. aeruginosa*, and infected mice were observed for 5 days (25).

### Statistical analysis

All statistical analyses were performed using SPSS v.26 (IBM Corp.). Data were assessed for normality using the Shapiro-Wilk tests and for homogeneity of variance using Levene’s test. For longitudinal IgG measurements (14th day OD, 21st day OD, and the phase 2 OD_14 [the second OD detected after 14 days]), a repeated-measures ANOVA with Greenhouse-Geisser correction was applied to account for within-subject correlations and violations of sphericity (Mauchly’s test *P* < 0.05). Between-group comparisons at each time point were analyzed using one-way ANOVA or Welch’s ANOVA when variance equality was violated. Post-hoc tests used the LSD method with Bonferroni correction. Effect sizes were reported as partial eta-squared (η²) for ANOVA and 95% confidence intervals for mean differences. Technical replicates (*n* = 5 per mouse) were averaged prior to analysis, with biological replicates consisting of seven mice per group. For survival analysis, we first compared categorical mortality rates at 72 h using Fisher’s exact test, then employed Cox proportional-hazards regression to estimate hazard ratios (HRs) with 95% confidence intervals (using the control group as reference), after confirming the proportional hazards assumption through Schoenfeld residual analysis (all *P* > 0.05). Technical replicates (*n* = 5 measurements per mouse) were averaged prior to statistical analysis, with biological replication consisting of seven mice per treatment group. All statistical tests were conducted as two-tailed analyses with a significance threshold of α = 0.05, and data are presented throughout as mean ± standard error of the mean (SEM) for continuous variables or median (interquartile range) for survival time measurements.

## RESULTS

### PCR amplification of *P. aeruginosa oprF* gene

**Fig 1:**
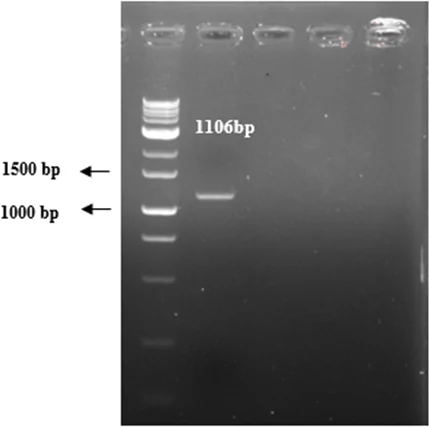
Conventional PCR amplification of *oprF* gene in *P. aeruginosa* PAO1 strain with the expected product length of 1,106 bp on a 1% agarose gel. L, 1 Kb PLUS DNA ladder; NC, negative control.

Amplification of *oprF* gene resulted in a single band with a size of 1,106 bp, which was within the expected product size range and confirmed amplification of the *oprF* gene (Fig. 1).

### Cloning of the *oprF* gene in the GetClone PCR Cloning Vector II

The purified PCR product was cloned into GetClone PCR Cloning Vector II. The existence of the *oprF* insert in the GetClone PCR Cloning Vector II was confirmed by colony PCR (see Fig. S1 at https://doi.org/10.6084/m9.figshare.32898893). Furthermore, DNA sequencing confirmed the identity of the *oprF* gene in the construct.

### Subcloning of the *oprF* gene in the pBluescript SK (+) vector

The *oprF* insert was cloned into the pBluescript SK (+) vector after digestion from the GetClone PCR Cloning Vector II (see Fig. S2 at https://doi.org/10.6084/m9.figshare.32898893). The *oprF* insert was cloned into pBluescript SK (+) vector. The presence of *oprF* insert in the pBluescript SK (+) vector was confirmed by colony PCR (see Fig. S3 at https://doi.org/10.6084/m9.figshare.32898893). The identity of the *oprF* gene in the construct was confirmed by DNA sequencing.

### Preparation and *in vitro* characterization of mRNA-OprF-LNP

**Fig 2:**
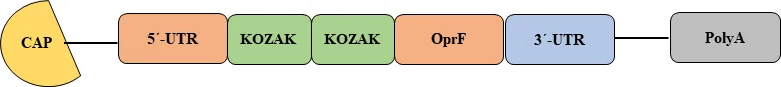
mRNA-OprF vaccine design and *in vitro* characterization. Schematic representation of the IVT mRNA structure. The OprF-mRNA construct consists of a 5′ cap, 5′ UTR, kozak, OprF, and 3′ UTR followed by a polyA tail.

**Fig 3:**
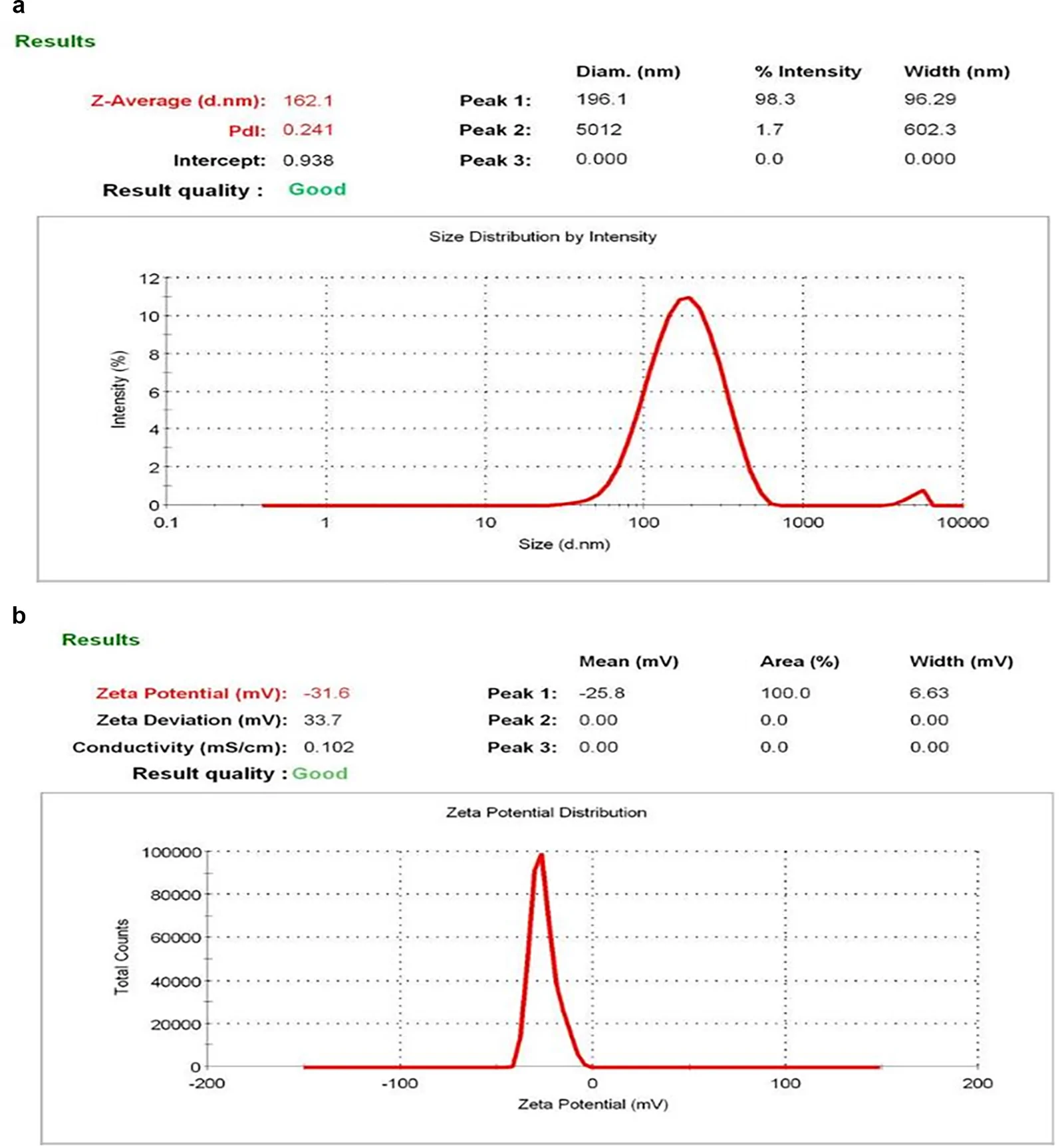
Physicochemical characterization and stability of the mRNA-LNP-OprF formulation. (**a**) Hydrodynamic particle size distribution of the lipid nanoparticles (LNPs), showing a mean diameter of 162 nm with a polydispersity index (PDI) of 0.241, indicating a monodisperse and uniform population. The single peak confirms the absence of significant aggregation. (**b**) Zeta potential distribution of the LNPs, showing a mean surface charge of −31.6 mV, which contributes to colloidal stability. Data are presented as the result of a representative measurement from three independently prepared LNP batches (*n* = 3). Stability studies confirmed no significant change in size, PDI, or zeta potential over time compared to the freshly prepared control.

In this study, we designed an mRNA vaccine encoding the full-length OprF protein in order to develop an effective vaccine against PA (Fig. 2). This protein contained a C-terminal 6×His tag for protein expression detection. The extracted pBlueScript II SK (+) plasmid containing the *oprF* gene was digested with the XbaI enzyme, and a linear plasmid with smooth ends was generated. After gel electrophoresis, the results confirmed the linearization of the plasmid (see Fig. S4 at https://doi.org/10.6084/m9.figshare.32898893). The desired mRNA was synthesized through T7 polymerase-mediated *in vitro* transcription. In order to deliver optimal mRNA, the synthesized mRNA was encapsulated into LNPs. According to our results, the size of the mRNA-containing liposomes determined by DLS was 162 nm. Furthermore, the PDI was 0.241, which showed an acceptable value and indicated the uniformity of the mRNA-LNPs size (Fig. 3a). Moreover, zeta potential measured for liposome containing mRNA was −31.6 mV (Fig. 3b). A single peak appearing in the DLS results indicates the absence of nanoparticle aggregation. Also, changes in particle size, polydispersity index (PDI), and zeta potential of LNPs were investigated using DLS. The results showed that there was no significant change in the particle size, polydispersity index (PDI), and zeta potential of LNPs compared to the control (freshly prepared) sample.

### Encapsulation efficiency (% EE) and mRNA loading

The encapsulation efficiency of mRNA-LNPs prepared in our study was higher than 99%. The measured values for mRNA loading were 95%.

### Cloning and expression of the OprF

For expression of OprF protein in *E. coli*, the *oprF* insert was removed from GetClone PCR Cloning Vector II using EcoRI and HindIII and subcloned into the pET-28a (+) expression vector (see Fig. S5 at https://doi.org/10.6084/m9.figshare.32898893). The existence of the *oprF* insert in the pET-28a (+) recombinant vector (pET-28a (+)-*oprF*) was also proven by colony PCR. Then, the identity and orientation of the *oprF* gene in the recombinant construct were confirmed by DNA sequencing. The recombinant plasmid pET28a (+)-*oprF* was transformed into *Escherichia coli* BL21 (DE3) as the expression host. Protein production was induced with IPTG (1 mM). OprF protein of approximately 43 kDa was detected after staining the SDS-PAGE gel with Coomassie Blue G-250. Then, the product was purified by Ni-NTA affinity chromatography (see Fig. S6 at https://doi.org/10.6084/m9.figshare.32898893).

### Investigation of humoral immune reactions in mice by ELISA method

**Fig 4:**
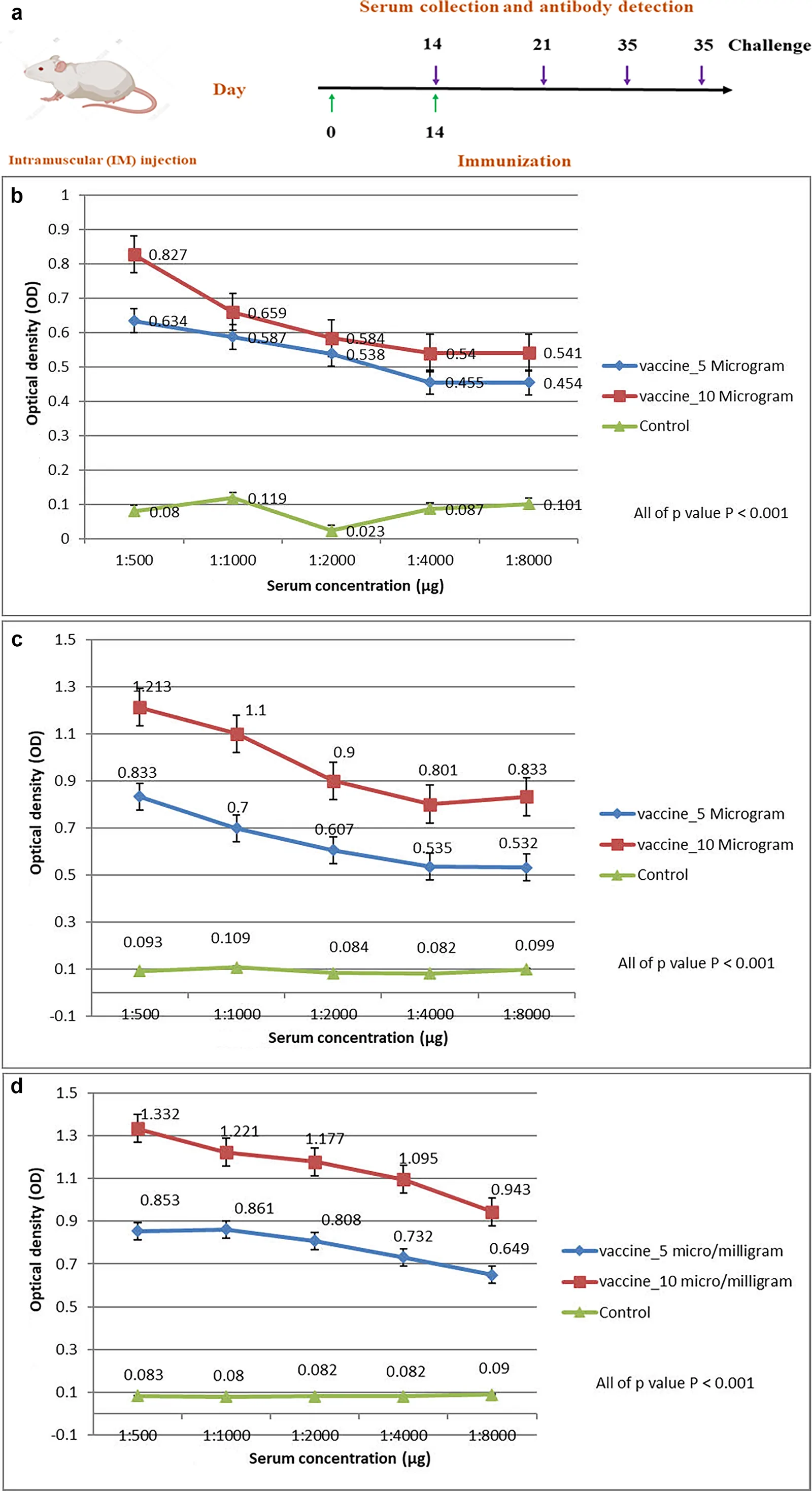
Investigation of the immunogenicity of the mRNA-LNP-OprF vaccine in mice. (**a**) Schematic representation of the immunization schedule and serum collection time points in BALB/c mice. (**b–d**) Serum IgG antibody responses measured by ELISA. Data are presented as mean optical density at 450 nm (OD450 ± SEM). The dashed horizontal line indicates the assay cutoff (OD450 = 0.15). (**b**) IgG levels measured 14 days after the first immunization (*n* = 7 mice/group). Data are from two independent experiments. (**c**) IgG titers measured 21 days after the first immunization at serial serum dilutions (*n* = 8 mice/group). The inset highlights responses at high dilutions (1:4,000–1:8,000). Data are pooled from three replicate experiments. (**d**) Specific IgG titers measured 14 days after the second immunization (*n* = 7 mice/group). Data are from two independent experiments. In panels **b–d**, statistical comparisons between groups (5 µg dose, 25 µg dose, and control) at each dilution were performed using one-way ANOVA (or Welch’s ANOVA when variance inequality was detected by Levene’s test), followed by LSD post-hoc tests with Bonferroni correction. ***P < 0.001 for both vaccine doses versus the control group at all dilutions.

As mentioned, to evaluate the immune response induced by mRNA encoding OprF, two groups of BALB/c mice (seven mice) twice with 25 μg (high dose) or 5 μg (low dose) of mRNA-LNP-OprF and control group with LNP were immunized intramuscularly. The interval between every injection was 2 weeks. Mice did not show any adverse clinical manifestations during the vaccination period. The blood samples were collected at the indicated time, and antigen-specific antibodies were detected by ELISA. ELISA plates were coated with purified recombinant OprF protein. OprF-specific IgG titer increased slightly 14 days after primary immunization, while it increased more 21 days after primary immunization (Fig. 4a and b). After the second immunization, OprF-specific IgG titer increased dramatically (Fig. 4c). The results of our study showed an increase in anti-OprF IgG antibodies in the serum of mice that received mRNA compared to the control group. In addition, injection of 25 μg of the high-dose mRNA vaccines was more favorable than 5 μg of the low-dose vaccine in increasing the level of anti-OprF antibodies (Fig. 4). The results of our study showed that mRNA-OprF-LNP immunization elicited antigen-specific humoral responses in BALB/c mice. The assumption testing results (see Table S1 at https://doi.org/10.6084/m9.figshare.32898893) demonstrate that IgG antibody levels were normally distributed (Shapiro-Wilk test, *P* > 0.05) for all groups except Group 3 (control) at the 14th day OD time point (*P* = 0.02), which required subsequent non-parametric analyses. Variance homogeneity (Levene’s test) was maintained at the 14th day OD and 21st day OD (*P* > 0.05) but was violated at phase 2 OD-14 (*P* = 0.04), necessitating the use of Welch’s ANOVA for this time point. These rigorous assumption checks ensured the validity of our parametric statistical analyses.

The repeated-measures ANOVA results (Table 2) revealed significant main effects of both time (F [1.42, 76.68] = 218.5, *P* < 0.001, η² = 0.82) and group (*F* [2, 18] = 412.7, *P* < 0.001, η² = 0.98), as well as a significant time × group interaction (F [2.84, 76.68] = 94.3, *P* < 0.001, η² = 0.74). These findings indicate that (i) antibody levels changed significantly over time, (ii) groups differed substantially in their immune responses, and (iii) the pattern of antibody development varied significantly between groups. The large effect sizes (η² > 0.7) suggest that these differences were biologically meaningful.

**TABLE 2:** Longitudinal IgG analysis (repeated-measures ANOVA)^*a*^

Effect	F (df)	*P*-value	Partial η²	Sphericity (Mauchly’s *P*)
Time	218.5 (1.42, 76.68)	<0.001	0.82	<0.001
Group	412.7 (2, 18)	<0.001	0.98	–^*b*^
Time × group	94.3 (2.84, 76.68)	<0.001	0.74	0.003

^*a*^ Partial eta-squared (η²).

^*b*^ –, *P*-values < 0.05 were considered statistically significant.

As shown in Table 3, between-group differences were significant at all time points (all *P* < 0.001), with Group 2 (10 μg) consistently showing the strongest immune response, followed by Group 1 (5 μg) and then Group 3 (all post-hoc comparisons *P* < 0.001). The 95% confidence intervals for the differences between Group 2 and Group 1 (ranging from [0.19, 0.23] at the 14th day OD to [0.42, 0.50] at the phase 2 OD_14) demonstrate progressively widening gaps in antibody levels between these groups over time. These results confirm that the experimental treatment in Group 2 produced significantly and consistently higher antibody responses than both the control and comparator groups.

**TABLE 3:** Between-group comparisons at each time point^*a*^

Time point	Test	F/W(df)	*P*-value	η²	Post-hoc (LSD)	95% CI (Group 2–1)
14th day OD	One-way ANOVA	412.7 (2, 18)	<0.001	0.98	2 > 1 > 3 (all *P* < 0.001)	(0.19, 0.23)
21st day OD	One-way ANOVA	587.2 (2, 18)	<0.001	0.98	2 > 1 > 3 (all *P* < 0.001)	(0.35, 0.41)
The phase 2 OD_14	Welch’s ANOVA	198.4 (2, 9.8)	<0.001	–^*b*^	2 > 1 > 3 (all *P* < 0.001)	(0.42, 0.50)

^*a*^ Partial eta-squared (η²).

^*b*^ –, *P*-valuse < 0.05 were considered statistically significant.

### Vaccination with mRNA-OprF-LNP protects against *P. aeruginosa* in infection models

**Fig 5:**
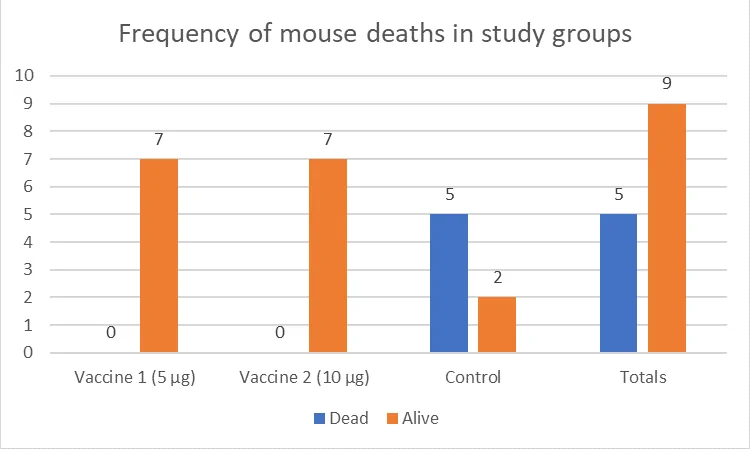
Survival outcomes following *P. aeruginosa* challenge in vaccinated and control mice. Mortality frequency in mice (*n* = 7 per group) over a 72-hour period after intraperitoneal challenge with 1 × 10⁶ CFU of *P. aeruginosa* strain PAO1. Both vaccinated groups (5 μg and 25 μg doses) demonstrated 100% survival (0/7 mortality), whereas the control group exhibited 71.4% mortality (5/7). Statistical significance was determined using Fisher’s exact test with Bonferroni correction; *P* < 0.001 for both vaccinated groups versus the control. Data are representative of two independent challenge experiments.

To determine whether mRNA vaccination could provide broad protection, immunized mice were challenged with standard strain PAO1 at the doses of 10 × LD_50_ 2 weeks after the second injection, and the survival was monitored for 5 days. All the mice in the control group died within 3 days after infection. In contrast, all the mice in the OprF-immunized groups survived when challenged with PAO1. The survival rate of infection models immunized with the mRNA-OprF was 100% (Fig. 5). As detailed in Table S2 (https://doi.org/10.6084/m9.figshare.32898893), active immunization with both vaccine candidates conferred a profound and statistically significant survival advantage following a lethal challenge with *P. aeruginosa* PAO1. The control group exhibited a mortality rate of 71.4%, with only 28.6% of mice surviving to the endpoint. In stark contrast, all immunized animals (100%) in both the 5 μg and 10 μg vaccine cohorts survived, resulting in perfect protection. The magnitude of protection is further quantified by hazard ratios, which indicate that vaccination reduced the instantaneous risk of death by 96% (HR = 0.04, 95% CI: 0.01–0.18) and 97% (HR = 0.03, 95% CI: 0.01–0.16) in the 5 μg and 10 μg groups, respectively (see Table S2 at https://doi.org/10.6084/m9.figshare.32898893).

## DISCUSSION

Nowadays, due to the growing trend of antibiotic resistance and the increasing number of MDR strains of *P. aeruginosa*, the effective treatment of infections caused by this pathogen has become one of the most challenging pathogens of health care units (28). In spite of 50 years of research and extensive efforts to discover an effective vaccine against *P. aeruginosa* infection, there is still no effective vaccine available to deal with infections caused by this pathogen (10). By retrospectively studying the history of PA vaccines, it seems that the reason for the limited success of these vaccines is probably the use of traditional vaccine technologies in the processes of these vaccines' development (29). The development of an effective vaccine is important to meet the challenges of treating this pathogen (30). The prominent features of mRNA vaccines are notable among nucleic acid vaccines. Nucleic acid vaccines have shown very favorable characteristics in terms of time and cost-effectiveness, as well as high safety and efficacy. Due to the mentioned cases, making a vaccine with mRNA technology against PA seems logical and necessary (31, 32). In addition, mRNA vaccines are made in a cell-free system (33). mRNA vaccines have been recognized as very promising therapeutic options with the emergence of the COVID-19 pandemic (34). Although, to date, this technology has been mostly used in viral infections and immunotherapies in the cancer era, it has rarely been used for bacterial infections (32). In this study, we designed an mRNA-LNP vaccine platform. After synthesis of the mRNA-LNP, we examined the antibody response in an animal model. It can be said that choosing the appropriate antigenic target for vaccine development is one of the most important challenges in developing vaccines for this pathogen (10). Among all the examined antigens of *P. aeruginosa*, OprF is considered an important antigen because it is expressed in most pathogenic strains and plays a basic role in its pathogenesis (35, 36). OprF, one of *P. aeruginosa* proteins, is anchored to the peptidoglycan layer and plays an important role in the full expression of virulence (37). OprF is highly conserved and serotype-independent, and several studies demonstrate the appropriate function of this protein as a suitable antigenic target in vaccine construction against PA. Also, it has been shown that active immunization with OprF can partially protect against infections with this pathogen (25, 38–40). Since the outer membrane protein OprF is expressed by all *P. aeruginosa* serotypes and is highly immunogenic, a vaccine containing this protein (IC43) has shown good immunogenicity and a high degree of efficacy against this pathogen. However, the rate of death and infection did not show a difference with the placebo group (14). In this study, the mRNA encoded the whole sequence of the OprF protein, which we expected in obtaining a suitable protective effect, while Wang et al. in 2023 developed an mRNA vaccine that included the OprF-I fusion protein of *P. aeruginosa* (14). So far, most research has focused on the C-terminal of the OprF protein as a vaccine candidate (41). It is noteworthy that, in the present study, complete mRNA synthesis was performed through *in vitro* enzymatic transcription (IVT). Today, LNPs are used as excellent delivery systems for mRNA vaccines (42). We used lipid nanoparticles (LNPs) as a suitable delivery platform for mRNA vaccines. It should be noted that, to date, there is no specific formulation approved by the FDA or European Medicines Agency for mRNA vaccine development, and various studies have used different formulations and lipids (31). In this study, we used the thin-film method as an uncomplicated approach in liposome production. We used lecithin lipid as a commercially available, ionizable, and inexpensive lipid, whereas other mRNA vaccines have used different formulations and expensive lipids (43, 44). The simplicity and reproducibility of this method and the ability to produce particles with the right size in this method were remarkable. The most important advantage of using this method is the production of lipid nanoparticles with a size below 200 nm, which enables their filtration through a 0.22 μm filter. Some studies have shown that smaller nanoparticles may not be immunogenic, while larger nanoparticles are more likely to induce an immune response (45). In the present study, we prepared nanoparticles with a diameter of about 132 nm and a PDI of about 0.241. Nanoparticles with a zeta potential in the ranges of ±40 to ±60 mV have good stability (46). The zeta potential of the particles in our study was −32 mV. This result was similar to the result reported in the study by Naderi Sohi et al., which showed a zeta potential of 3 mV (26). In addition, sterile filtration of the final product is also possible through this method. In all stages of the animal phase, the vaccine was well tolerated, and no local adverse reactions were observed after injection. The survival percentage of mice from the time of inoculation to before the challenge was 100%. Similar to the study of Wang et al. in 2023, the vaccine synthesized in our study induced humoral immune responses in mice as a non-human host (14). The second group of vaccines that received a higher dose (25 µg) of the vaccine compared to the first group (5 µg), which received a lower dose of the vaccine, showed a higher specific antibody level, which was not unexpected. A number of studies have investigated the mechanism of *P. aeruginosa* clearance, but it is still unclear what type of immune response is important for a vaccine (10). A number of studies have shown the necessity of humoral immune responses in preventing PA infections (47–50). The vaccine made in this study was able to induce a humoral immune response in the tested mice. This humoral immune response induced by the mRNA-OprF-LNP vaccine in our study is in line with the study of Wang et al. in 2023, which showed that humoral immunity can provide protection against infections with this pathogen (14). Finally, during the current study, the efficacy of the prepared vaccine was evaluated in mice. Challenge tests are important indicators to evaluate the effectiveness of vaccines (51). In our study, it was shown that our vaccine can provide a good protective effect to vaccinated mice. In the study by Wang et al. in 2023, the results of the mouse challenge showed that the vaccine developed in their study had a 100% protective effect (14).

### Conclusion

In this study, a vaccine of mRNA encoding the complete sequence of outer membrane protein F in *P. aeruginosa* was synthesized and encapsulated within lipid nanoparticles. To the best of our knowledge, this study was the first study in Iran to develop an mRNA vaccine against *P. aeruginosa*. Although we used simple production methods, the prepared mRNA-LNP vaccine appeared to induce a suitable level of OprF protein-neutralizing antibodies against *P. aeruginosa* in mice. However, further studies on cellular immunity in vaccinated BALB/c mice seem necessary, and these studies are ongoing in our laboratory.

## Data Availability

All data are available in the main text or supplemental information. Additional information is available from the corresponding author upon request.
